# Theoretical and Methodological Approaches to Ecological Changes, Social Behaviour and Human Intergroup Tolerance 300,000 to 30,000 BP

**DOI:** 10.1007/s10816-020-09503-5

**Published:** 2021-02-03

**Authors:** Penny Spikins, Jennifer C. French, Seren John-Wood, Calvin Dytham

**Affiliations:** 1 Department of Archaeology, Archaeology PalaeoHub, University of York, York, YO10 5DD UK; 2 Department of Archaeology, Classics, and Egyptology, University of Liverpool, Liverpool, L69 7WZ UK; 3 York Cross-disciplinary Centre for Systems Analysis (YCCSA) Internship Programme, University of York, York, UK; 4 University College London, London, UK; 5 Department of Biology, University of York, York, YO10 5DD UK

**Keywords:** Modern human behaviour, Tolerance, Social connectivity, Agent-based model, Environmental change, Palaeolithic, Self-domestication

## Abstract

Archaeological evidence suggests that important shifts were taking place in the character of human social behaviours 300,000 to 30,000 years ago. New artefact types appear and are disseminated with greater frequency. Transfers of both raw materials and finished artefacts take place over increasing distances, implying larger scales of regional mobility and more frequent and friendlier interactions between different communities. Whilst these changes occur during a period of increasing environmental variability, the relationship between ecological changes and transformations in social behaviours is elusive. Here, we explore a possible theoretical approach and methodology for understanding how ecological contexts can influence selection pressures acting on intergroup social behaviours. We focus on the relative advantages and disadvantages of intergroup tolerance in different ecological contexts using agent-based modelling (ABM). We assess the relative costs and benefits of different ‘tolerance’ levels in between-group interactions on survival and resource exploitation in different environments. The results enable us to infer a potential relationship between ecological changes and proposed changes in between-group behavioural dynamics. We conclude that increasingly harsh environments may have driven changes in hormonal and emotional responses in humans leading to increasing intergroup tolerance, *i.e.* transformations in social behaviour associated with ‘self-domestication’. We argue that changes in intergroup tolerance is a more parsimonious explanation for the emergence of what has been seen as ‘modern human behaviour’ than changes in hard aspects of cognition or other factors such as cognitive adaptability or population size.

**Supplementary Information:**

The online version contains supplementary material available at 10.1007/s10816-020-09503-5.

## Introduction—Approaches to the ‘Modern Human Transition’

Of all the key transitions in human evolution it is that which occurred between 300,000 and 30,000 years ago—the ‘modern human transition’*—*which is the focus of the most intense debate (Högberg and Lombard, this volume)**.** It is during this period that we see the emergence of our own species *Homo sapiens*, otherwise referred to as anatomically and cognitively modern humans (ACMH).

Whilst there remains a consensus that after 300,000 years ago, and following the spread of modern humans out of Africa, the range and frequency of key elements of ‘modernity’ increase (French 2018), the broader mechanisms by which new biological forms of hominin and new types of technological and social behaviour emerge remain poorly understood (d’Errico and Banks 2013; Moncel and Schreve 2016). Modern human behaviour (defined as behaviours that indicate modern-level linguistic and cognitive abilities and identified archaeologically through the presence of, among others, deliberate burials, complex lithic and hafting technologies, personal ornamentation, pigment use and ‘symbolic’ art and artefacts; Henshilwood and Marean 2003; Mellars 2007) is far from unproblematic as a concept (Ames *et al.* 2013), and many elements of such behaviour were also exhibited by archaic humans (*e.g.* Zilhão *et al.* 2010; Joordens *et al.* 2015; Hoffmann *et al.* 2018; Kissel and Fuentes 2018). Furthermore, prosocial motivations and behaviours, including care for the ill and injured (Spikins *et al.* 2019), and collaborative hunting practices and food sharing (Domínguez-Rodrigo *et al.* 2014; Agam and Barkai 2016; Faurby *et al.* 2020), emerged relatively early in human evolution. Nonetheless, it is largely after 300,000 years ago that many complex social and cultural behaviours became widespread.

Certain particularly interesting patterns of change are evident in human social behaviours in Africa 300,000–30,000 years ago. Alongside increased ecological variability in East Africa around 300,000 BP, we see evidence of increased raw material transfer distances (Potts *et al.* 2018), indicating changes in patterns of group and intergroup mobility. From typically local raw material transfer distances of around 5 km, we see new movements of obsidian of around 25 to 50 km—and up to 95 km in certain cases—implying interactions with neighbouring groups (Brooks *et al.* 2018). Middle Stone Age populations in the Kalahari also imported preferred silcrete raw material from up to 295 km, particularly during drier periods (Nash *et al.* 2013, 2016), well beyond the transfer distances typically recorded in previous periods. Greater patterns of large-scale regional mobility both within Africa and beyond are also evident from genetic data (Timmermann and Friedrich 2016; Lamb *et al.* 2018; Petraglia *et al.* 2019; Rito *et al.* 2019).

Important anatomical changes associated with the emergence of anatomically modern humans also occurred during the same period, with so-called craniofacial ‘feminisation’ drawing the most attention (Cieri *et al.* 2014). From around 300,000 years ago, certain populations in Africa display traits such as a reduction in brow ridges and other changes in facial form, as well as increased gracility associated with anatomically modern humans (Stringer and Galway-Witham 2017), with populations at Jebel Irhoud in Morocco dating to around 315,000 years ago being a particularly notable example (Hublin *et al.* 2017; Richter *et al.* 2017). Whilst archaic forms continued to be represented, crania such as that from Omo 1, dated to around 195,000 years ago or Herto, dated to 165,000-100,000 years ago are considered modern in appearance (Klein 2019). This was a period of both marked behavioural change and marked physiological and anatomical change.

These archaeological and anatomical changes were set against a backdrop of marked ecological challenges. Across the whole continent, the expansion and contraction of the Sahara basin structure and variable topography provided a unique environment (Foley 2018) in which distinct subdivided populations seem to have emerged and periodically connected (Scerri *et al.* 2018; Galway-Witham *et al.* 2019). Both southern and eastern Africa were key to the emergence of modern humans (Rito *et al.* 2019). Increasing aridification from half a million years ago in East Africa placed particular pressures on the survival of many mammalian species and is associated with mammalian extinctions between 500 and 400,000 years ago (Owen *et al.* 2018). Alternating periods of arid and wetter conditions also affected southern African environments, placing particular pressures on human populations in arid periods and prompting dispersions along wetter corridors (Simon *et al.* 2015; Kutzbach *et al.* 2020). Whilst the precise conditions under which our species emerged remain unclear and much debated, distinctively spatially and chronologically variable—and often increasingly resource poor—environments appear to have been key to the complex patterns of evolutionary change taking place within both archaic and modern humans.

**Fig. 1:**
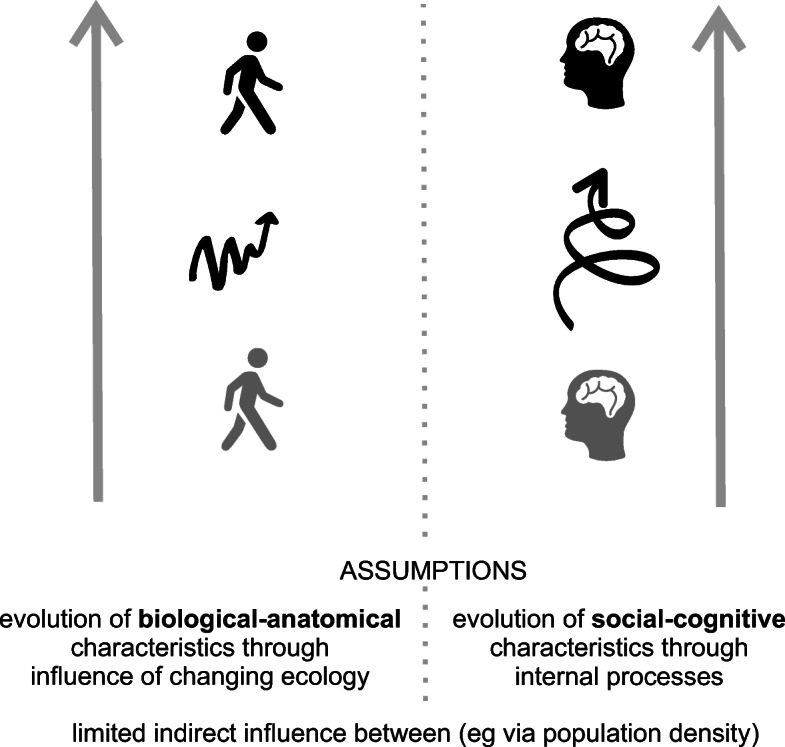
Simplified graphical illustration of commonly assumed distinctions and relationships between evolutionary processes affecting the evolution of the human mind and body as separate processes affected by differing influences. Left: representation of assumed evolution of body shape through interactions with the environment, right: representation of assumed evolution of mind through internal social processes

The mechanisms by which these ecological changes might lead to such notable changes in anatomy, physiological and behaviour remain to be explored. A particular challenge lies in understanding the relationship between biological/anatomical and/or social/cognitive change, and how these relate to ecological context. All too often traditional disciplinary boundaries, alongside preconceptions about how evolutionary processes *ought to work*, further a distinction between changes in body (biological/anatomical change) assumed to be driven by ecological changes and changes in mind (social/cognitive change) assumed to be driven by internal social processes (Fig. 1).

## Ecological Changes and Selection Pressures on Social Tolerance

An understanding of how ecological contexts influence changes in social-emotional dispositions may provide a pathway to link ecological-biological and social-cognitive approaches and contribute additional insights into the nature of key transformations occurring 300,000–30,000 years ago.

Evolutionary transformations in emotional dispositions and responses are likely to have played an important role in key transitions in human evolution (Decety *et al.* 2012; Spikins *et al.* 2019; Marsh 2019; Spikins 2021). Variations in oxytocin responses, for example, have a notable influence on caring behaviours in modern human populations (Marsh 2019), undergo significant changes in human evolution (Theofanopoulou *et al.* 2018) and are implicated in food sharing (Wittig *et al.* 2014), care for injured adults (Spikins *et al.* 2019) and teaching and learning in other species (Thornton and McAuliffe 2006). The transition into a new human niche involving greater levels of carnivory from around two million years ago (Domínguez-Rodrigo *et al.* 2014) is likely to have involved changes in collaborative emotional dispositions, including those affected by oxytocin, that facilitated food sharing, shared infant care and care for vulnerable and injured adults, much as is seen among social carnivores.

Emotional dispositions also play a key role in social connectivity at a regional scale. Emotional reactions to ‘outsiders’ are influenced by hormonal responses affecting approach behaviours (*i.e.* friendliness) through hormonal influences on fight or flight responses (affected by hormones such as cortisol) and willingness to explore (affected by hormones such as dopamine; Wilkins *et al.* 2014). Key changes in these hormone systems occurred over the last 300,000 years (Theofanopoulou *et al.* 2017) and have also been implicated in the evolution of fully modern language (Thomas and Kirby 2018). Whilst it would be foolish to suggest that anything as complex as human regional social interactions is just about biology, the influence and constraints of emotional responses play an important role even in modern contexts (Sapolsky 2017).

### Ecological Selection Pressures on Intergroup Tolerance

The relationship between ecological changes and selection pressures on intergroup tolerance may have played a significant role in the changes in social connectivity and mobility that occurred 300,000–30,000 years ago. The relationship between ecology, resource distributions and intergroup tolerance in mammals in general, and in primates specifically, provide useful insights.

Even though unfamiliar individuals are typically a threat to territories or resources, there are several factors which can promote rather than constrain tolerance towards unfamiliar or ‘outgroup’ individuals. The most obvious and important factor is access to resources. Tolerance enables exploitation of resources at boundaries whilst avoidance or aggression makes such exploitation impossible. The friendly interaction at boundaries recorded in bonobos (*Pan paniscus*) facilitates exploitation of boundary resources such as fruiting trees as well as small prey, for example (Tan and Hare 2013; Tan *et al.* 2017; Hare and Yamamoto 2017; Lucchesi *et al.* 2020). Bonobos from different groups will willingly share food with non-group members and have been observed actively sharing with other groups at boundaries (Tan *et al.* 2017). In ecological contexts, where resources are highly clustered and critical for survival, tolerance may be particularly key to enabling access (Pisor and Surbeck 2019).

**Fig. 2:**
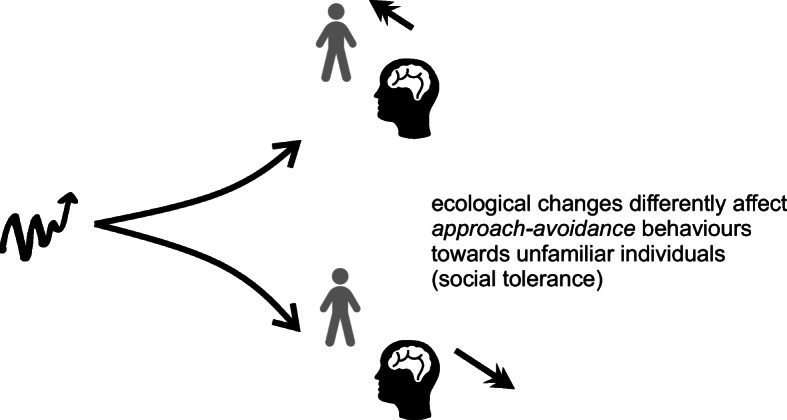
Graphical illustration of how ecological changes affect tendencies to approach-avoidance behaviours towards unfamiliar individuals through evolved hormonal responses affecting social tolerance. Ecological changes (left) can have different evolutionary effects on brain and physiology from promoting more tolerant behaviours (upper right) to promoting less tolerant behaviours (lower right)

The advantages which tolerance may bring to resource exploitation is not the only factor promoting tolerant intergroup interactions. Other factors include the following: the potential for gathering information before the transfer of individuals within mating networks; increased opportunities for extra group meeting and collaborative defence (Pisor and Surbeck 2019). Collaboration between unrelated colonies has even been recorded in eusocial ants as a means of collaborative predator defence (Robinson and Barker 2017). Clearly, ecological changes affecting resource availability and the distribution of resources, as well as other factors such as predation will influence selection pressures on tolerant, rather than avoidant, or aggressive, reactions to ‘outsiders’ (Fig. 2).

Archaic humans would have been particularly vulnerable to these changes due to their dependence on several types of resources; not only plant and animal foods but also raw materials for tool manufacture and other resources such as medicines (Hardy 2018). Modern ethnographically documented hunting and gathering populations demonstrate a high degree of intergroup interactions (Bird *et al.* 2019) and dependence on intergroup transfers (Pisor and Surbeck 2019). Intergroup collaboration allows access to widely distributed resources, such as salt, medicines and raw materials for toolmaking (Pisor and Surbeck 2019) and buffers against resource unpredictability and shortfalls (Wiessner 2002; Dyble *et al.* 2016). The Ju’hoansi ‘hxaro’ network—a system of distant allies able to provide support in times of resource shortfall—is perhaps the best-known ethnographic example of how intergroup tolerance and collaboration foster survival (Wiessner 2002). Many other examples also exist. In Tierra del Fuego, beached whales are exploited by different communities who reciprocate the opportunity by alerting others and allowing entry into their territory (Santos *et al.* 2015).

Attention has focused on the significance of multilevel networks in human evolution (Grove *et al.* 2012; Layton *et al.* 2012). However, a focus on changes in social tolerance *between* foraging or kin groups may be a more useful theoretical approach, particularly given that evidence for high levels of inbreeding (discussed below) is difficult to reconcile with what we know of multilevel networks. Whilst we have assumed that the evolutionary origins of regional networks of connectivity lie predominantly in an increasingly complex cognition, physiological changes influencing social behaviour may have played a far more significant role than has previously been considered.

### Intergroup Tolerance in Archaic Humans

There was almost certainly some level of regional population connectivity among archaic humans (*e.g.* Greenbaum *et al.* 2019), although evidence suggests that this social connectivity was subject to notable constraints. For example, evidence from skeletal abnormalities (Ríos *et al.* 2015, 2019; Trinkaus 2018) and genetics (*e.g.* Castellano *et al.* 2014) suggest high rates of inbreeding throughout the Lower and Middle Palaeolithic which would be unlikely to have occurred where social groups were fluid and connected. Across the archaic world, there were limited connections beyond home ranges until at least 500,000 years ago (Marwick 2003; Layton *et al.* 2012). In Eurasia, the long genetic history of close interbreeding (equivalent to the half-sibling level) seen in the genome of the Altai Neanderthal (Prüfer *et al.* 2014), contrasts with the evidence for the ACMH Sunghir burials II, III and IV, whose genome sequences indicate exogamous mating practices (Sikora *et al.* 2017). Nonetheless, connections of some kind over long distances existed. In Middle Palaeolithic Europe, there are rare examples of long distance material movements (Féblot-Augustins 1999), and even rarer examples of distant raw materials predominating where local materials are unsuitable as reported by Spinapolice (2012) in southern Italy. However, as a whole, there is little evidence for frequent social interaction between groups. The evidence for longer distance movements outside of a group’s typical range is consistent with what we might expect when external social connections were uncommon (Djindjian 2012), perhaps limited to movements around mating patterns (which may have been constrained by patrilocality; see Lalueza-Fox et al. 2011).

Even what we might consider as the first stage of regional intergroup connectivity—resource exploitation at boundaries—is not always evident. In the Middle Palaeolithic of the Levant, detailed studies of the transport of flint materials to the site of ‘Ein Qashish’ even suggest potential borders between groups where resources remained unexploited (Ekshtain et al. 2014, 2017; Hovers 2018). We can reasonably assume that archaic groups were capable of the kind of intergroup or landscape scale interactions recorded in bonobos (*i.e.* exploitation of resources between groups and some sharing of resources), not only on the basis of common ancestry but also on the basis of evidence from raw material transfers. However, the level of social tolerance which we often assume characterised human societies for much of our evolutionary past—frequent social connection and access to complementary resources as well as mating opportunities—is far more elusive than we might expect.

There is little doubt that transformations in connectivity 300,000–30,000 years ago significantly changed regional social relationships, laying the basis for fluid social and biological connections to emerge, as well as regular aggregations, and the spread of new innovations and ways of doing things (Gamble 2009; Coward 2015; French 2016, 2018). Physiological changes in response to the influence of changing ecology on selection pressures towards intergroup social tolerance are likely to have played a key role in these transformations. Whether ‘self-domestication’ is an appropriate term to apply to changes in the human evolutionary past or not remains debated (see Sánchez-Villagra and van Schaik 2019). Nonetheless, it is clear that between 300,000 and 30,000 years ago, there were transformations in physiology and anatomy in emerging modern human populations associated with changes in hormone function and which are broadly associated with increased tolerance (Theofanopoulou *et al.* 2017). Analogies have been drawn between the changes observed in humans and those seen in other primates (such as distinctions between common chimpanzees and bonobos) and other species less closely related to humans (such as wolves and free ranging dogs).

Here, we investigate the mechanisms behind these changes in social disposition which are often generalised within the term ‘self-domestication’. To illustrate potential methods to better understand such changes, we develop an agent-based model (ABM) to simulate the potential effects of ecological changes on intergroup tolerance in archaic humans. We simplify two different populations within the broad classification as being ‘avoidant’ and ‘tolerant’ in order to compare these different strategies in differing ecological contexts.

## Method

### Model Overview

We use a spatially explicit, agent-based model to simulate individuals attached to groups (or ‘bands’ within modern ethnographic contexts; Hill *et al.* 2014) of hunter-gatherers. Agent-based models are a widely used tool for investigating complex systems (Railsback and Grimm 2019). They have long been used in archaeology to reveal how individuals interact with each other and their environment to produce emergent patterns (reviewed by Premo 2006; Cegielski and Rogers 2016; Romanowska *et al.* 2019). It has been shown that prey depletion across a landscape with interacting individuals is best addressed using a simulation model (Křivan and Eisner 2003). Brantingham (2003) used a model of individuals moving around a spatially heterogeneous landscape encountering, collecting and processing resources to provide a null model of the diversity of stone sources that would be found in a toolkit.

Here, we model individual humans moving around a dynamic landscape hunting resources (similar to Janssen and Hill 2014, but with more abstract animal populations). Our focus is on the effect of the nature of intergroup interactions, and whether food resources are shared when groups meet. Individuals foray from their group foci to acquire resources and to interact with other groups, and they also age and may reproduce. The model is implemented in C# and compiled and run on a PC using Microsoft Visual Studio 2019.

Like every model, this simulation cannot represent the full extent of all social interactions among archaic humans; therefore, it simplifies some of these aspects to allow us to explore key questions. We use a series of assumptions based on a simplification of what is known about the social behaviour of archaic humans:

- We assume that archaic humans belong to groups distributed across a landscape, and that these groups can move around and interact with other groups
- Groups may interact, with the probability of interactions higher when group foci are closer (*i.e.* that each group is not seeking each other out but interacting randomly)
- Social interactions may be ‘avoidant’ or ‘tolerant’, with the latter allowing for potential transfers of resources from a group with excess resources to one with a deficit (resource sharing)
- Resources (in this case hunted food, though foraged plant foods would function in the same way) are tracked in landscape cells. Food is needed for maintenance and excess food is needed for successful reproduction
- Animal populations increase following logistic growth and successful hunting removes animals from the landscape and adds food to a group’s supply
- Individuals age and suffer age-dependent mortality, and mature females can reproduce (when the group has sufficient resources)

Our use of ‘tolerance’ in this context implies a positive interaction with members of other groups, leading to the possibility of resource transfers to those in need from those with available resources, in accordance with sharing as observed in modern ethnographically documented contexts (Lavi and Friesem 2019; Spikins 2019).

### Model Operation

#### Initialisation

At initialisation there are 160 group foci placed randomly in continuous space within the landscape. The starting population of humans is 3000 individuals randomly assigned to a group and starting at the group focus. Individuals have a random age (1 to 50) and sex (even chance) assigned at the start of the simulation. Animal populations are set independently for each grid cell and initially have a random value (1 to 100).

#### Model Flow

There are several phases within a model year. Further details of these phases are given in the Supplementary Materials.

##### Hunting

Individuals each start from their group focus point and, if old enough to hunt, take a series of step moves with hunting attempted at the end of each step. A successful hunt adds a unit of resource to the group’s stock.

##### Intergroup Interactions

Pairs of groups are selected at random and may meet for an intergroup interaction where, if tolerant, resources may be exchanged.

##### Maintenance

Individuals eat food from the group’s supply for subsistence.

##### Ageing, Birth and Death

All individuals age each year and there is an age-specific probability of death. Adult females in groups with excess resources may have offspring.

##### Group Fission and Loss

Groups of size 50 and above split, those below size 4 are lost.

##### Animal Population Growth

See Supplementary Materials.

#### Elements and Variables of the Model

The elements and variables used in the model are described in Table 1.

**Table 1:** Explanation of elements and variables used in the model

Element	Explanation
Landscape	The landscape is represented by a regular grid of 100 × 100 landscape cells (the side length referred to as a ‘grid unit’). Each landscape cell supports an independent animal population.
Individual	An individual human located in continuous space within the landscape. An individual’s sex, age and group affiliation is tracked.
Group	Individuals are assigned to groups. Each group has a focal point or ‘camp’, located in continuous space, that remains fixed for a season. Groups are assumed to pool hunted resources and successful hunting adds to the group’s stock.
Hunting	All individuals older than 10 are assumed to move in forays through the landscape and hunt resources (detailed description in supplementary materials and see flowchart in supplementary section).
Maintenance and starvation	Each group loses 1 unit of food for each group member to provide subsistence. If there is insufficient food to cover this maintenance, individuals may starve (detailed description in supplementary materials).
Birth and death	Females between ages 16 and 39 have offspring if there is sufficient food after maintenance to cover the birth cost. There is age-dependent death applied following (Gurven and Kaplan 2007; Hill *et al.* 2007; Kelly 2013) in addition to death from starvation.
Group loss and group fission	Any group with fewer than four members is dissolved, and all remaining group members are assumed to have died. Any group with 50 or more members will split into two. At group fission, all individuals in the current group are randomly assigned to one of the two daughter groups. One group will have its focus in a new location (details in supplementary materials).
Animal population growth	Each landscape grid square has an independent animal population and at the end of each year, populations can increase following logistic growth (details in supplementary materials).
‘Harshness’ of environment	Harshness of the environment is varied by varying the cost of births. Here we use a range of costs of reproduction from 26 to 35.
Tolerance (potential for resource transfer)	Within a simulation run, all groups are either ‘avoidant’ or ‘tolerant’. If groups avoid each other, no food will be transferred, and when groups are tolerant, food will be transferred (shared) if one has a surplus and the other a deficit.
Storage of resources	There is no long-term storage of resources and groups start each hunting season with no stored food.

### Model Realisations

For each model realisation here, we focus on the total population size as a measure of success. In all simulations, the population size reported is the mean total population within a realisation between timesteps 901 and 1000.

Populations within a simulation are either ‘avoidant’ where there are no intergroup interactions (other than indirectly through exploitation competition) or ‘tolerant’ where positive intergroup interactions (food sharing) are possible. We vary the ‘harshness’ of the environment by changing the cost of offspring from 26 (benign) to 35 (harsh). There are 200 replicate simulations of each tolerance/environment combination.

We then repeated the simulations with temporal environmental heterogeneity. This was achieved by adding variation in the cost of reproduction between years to simulate a mix of good and bad years with the same mean. Each year we added a value to the cost of reproduction value drawn as a random uniform integer (− 7 to 7), mean 0, standard deviation of 4.7. Variation was added independently each year and there was no temporal autocorrelation.

**Fig. 3:**
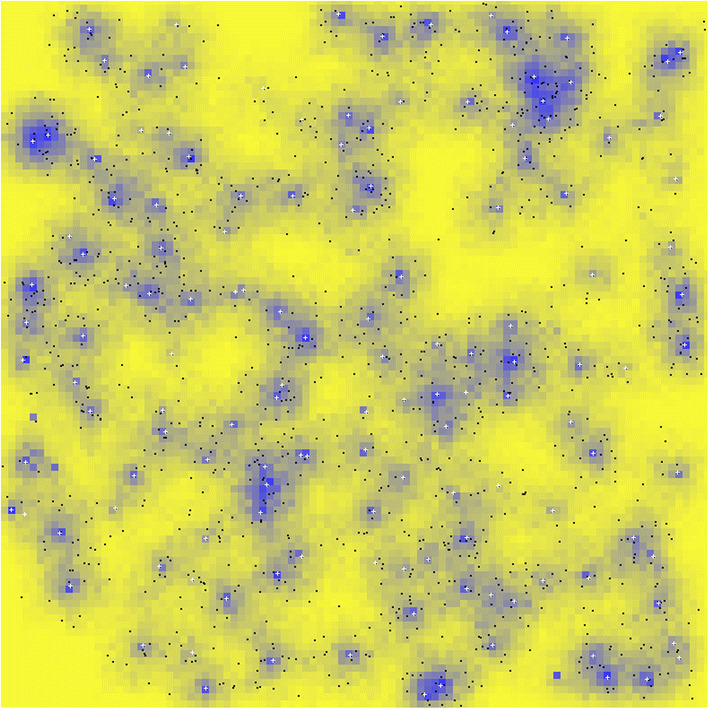
A snapshot of the model in action. Blue dots are individual foragers, and white crosses are group foci or ‘camps’. Dark blue to yellow shading in landscape cells indicates the level of available resource from low to high. The effect of depletion of resources near camps is clear

An illustration of the model in operation is shown in Fig. 3.

## Results

The model outputs allow us to make observations about the advantages or disadvantages of strategies of tolerance or avoidance of other groups under different environmental conditions.

**Fig. 4:**
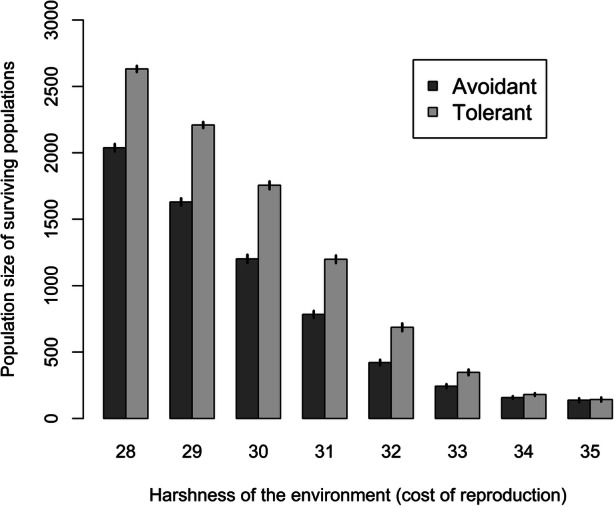
A range of costs of reproduction with avoidant (dark tone, left column) and tolerant (light tone, right column) simulations. Data shown are the mean population size at the end of the simulation from up to 200 replicates for each bar (fewer in more harsh environments where there are extinctions). Error bars show 95% confidence intervals

Unsurprisingly, the harshness of the environment has a notable effect on the population size (Fig. 4), with harsher environments supporting smaller populations. Intriguingly, this effect is much more pronounced for avoidant than tolerant strategies—sharing food resources across borders is advantageous, leading to higher population density and greater probability of survival. Sharing can still be costly nonetheless, and interestingly the benefits of sharing become less evident in the harshest environments as the costs of sharing become more significant in relation to resources required for immediate survival.

**Fig. 5:**
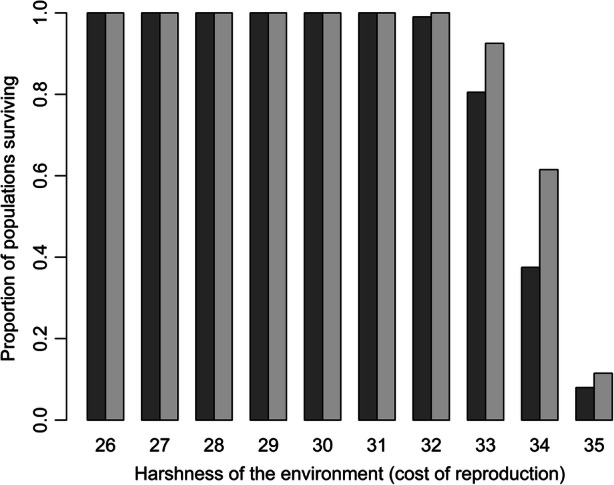
Proportion of simulations with population over zero at the end (*i.e.* survival). Two hundred simulations for each bar with avoidant (dark grey) and tolerant (light grey) simulations

The relative probability of populations surviving or failing to secure enough resources for survival under different strategies of avoidance or tolerance to other groups also shows interesting patterns. In benign environments, all populations persist, but again, unsurprisingly, harsh environments (where reproduction is costly) reduce population survival (Fig. 5). However, interestingly, tolerant populations (which are able to share resources) are less affected by increasingly harsh environments—a tolerant population not only has a higher population size than an avoidant population (Fig. 4) but also a tolerant population is more likely to survive in a harsh environment, and therefore less likely to become locally extinct (*i.e.* where a total population falls to zero) (Fig. 5).

Statistical analysis supports these observations. A two-way ANOVA with population size as the response variable and tolerance and cost of reproduction as predictors shows strongly significant effects of both factors. There is also a highly significant interaction between predictor factors. Effect sizes, as measured by *η*^2^, for the main effects are 0.94 for cost of reproduction, 0.27 for tolerance and 0.01 for their interaction, with all *p* values < 0.001, although significance levels for simulation models should be treated with some caution (White *et al.* 2014). To investigate the form of the interaction between tolerance and cost of reproduction (environmental harshness), we expressed the results as tolerant population size/avoidant population size. Tolerance (food sharing) has a positive effect (ratio > 1) throughout, but the scale of the effect varies with environmental harshness.

**Fig. 6:**
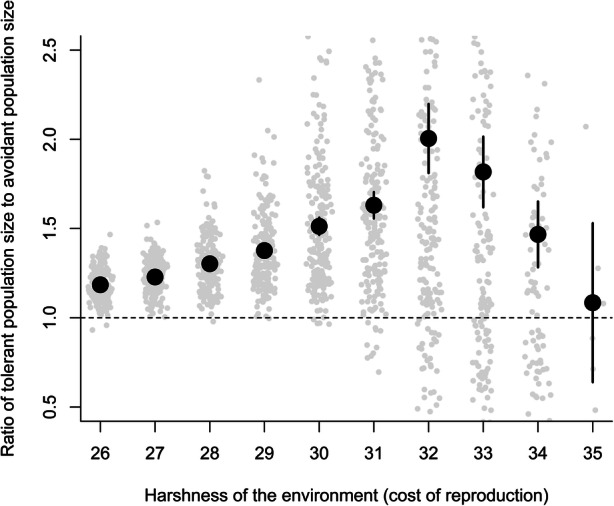
Population size of tolerant simulations/avoidant simulations, means are filled black circles. A value of 1 (shown with a dashed horizontal line) will result if there is no effect of resource sharing, values over 1 indicate tolerant populations are larger, a value of 1.5 showing 50% larger. Two hundred pairs of simulations (1 avoidant, 1 tolerant) were run for each level of environmental harshness, and raw data for the ratio in each pair are shown in light grey circles. Ratios are only available when both populations in the pair of simulations persisted to the end of the simulation. 95% confidence intervals are shown (note that for low values of environmental harshness, these are within the circle showing the mean)

As we see from Fig. 6, whilst tolerance is a generally advantageous strategy, this advantage is most pronounced where environments are neither extremely benign (where sharing becomes less necessary for reproduction and survival) nor extremely harsh (as the costs of sharing become more significant in relation to the resources needed to reproduce and survive).

**Fig. 7:**
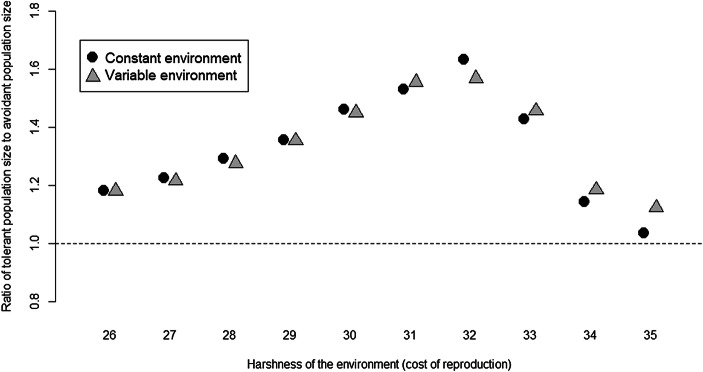
Interaction between tolerance and cost of reproduction for a constant environment (black circles) and variable environment (grey triangles). Each point is the mean tolerant population size/mean avoidant population size (dashed horizontal line indicates equal population sizes for tolerant and avoidant strategies). There were 200 realisations of each strategy for each level of cost of reproduction for both constant and variable environments. All realisations below cost of reproduction 32 persisted. Simulations where the population died out were discarded (see Fig. 5)

Figure 7 shows the effect of adding environmental variability through interannual variability in cost of reproduction. It confirms the pattern in Fig. 6, indicating a clear peak for the benefit of food sharing at harshness of 32, but that the drop from there as conditions become harsher is larger in a constant environment than a variable one. Tolerance is even more beneficial to overall population success in a harsh (over level 33) and variable environment than when environments are more productive and stable. However, this pattern is much less pronounced than the overall effect within harsh rather than benign environments.

## Discussion

### The Relationship Between Ecology and Human Intergroup Tolerance

The model outlined here considers the implications of tolerant or avoidant strategies on forager success and survival when encountering other groups under different environmental conditions.

Our simulations demonstrate that intergroup tolerance, allowing the exchange or sharing of resources between groups, has a significant effect on population survival in ‘harsh’ or difficult environments. Populations which share resources are likely to be more successful (*i.e.* increase in population) and are more likely to survive harsh environments where extinctions occur than those populations which do not share across borders. This finding supports arguments made on the basis of ethnographically documented resource transfers at times of famine (see for example Wiessner 2002).

We also demonstrate novel patterns within the broader advantages of tolerance. Firstly, the effects of intergroup tolerance are most significant in moderately harsh environments. In the harshest environments, population density becomes too low to support interactions (the cost of interactions are high compared to the resources needed to survive, groups have little resources to spare to share and survival becomes critical). In the most benign environments, however, the benefits of sharing become marginal (as resources for reproduction and survival are not threatened). Secondly, overall harshness has a far greater effect on the selection pressures promoting social tolerance than ecological variability, though ecological variability does have some effect.

The most significant finding in terms of broader debates over changes between 300,000 and 30,000 BP is that tolerance towards other groups and intergroup collaboration becomes advantageous as environments become harsher (though in extremely harsh environments it becomes difficult to maintain the level of intergroup contact required to make collaboration possible) and tolerance also becomes more advantageous as environments become more variable. Although to date there has been some understanding of why intergroup collaboration might make communities more resilient, there has been little understanding of the ecological factors which might influence this, or the limitations of collaborative strategies in certain ecological contexts. This finding therefore provides some support for suggestions that environmental variability may have played a role in social changes in recent human evolution (Potts 2013; Potts *et al.* 2018). However, the effects of environmental variability on the selective advantages of intergroup tolerance are much less pronounced than the overall effect within harsh rather than benign environments. This result is perhaps surprising given the emphasis in the literature on environmental variability as a driver for human evolutionary changes rather than environmental harshness *per se*. Whilst variability is clearly an influence on selective pressures, the potentially elevated significance of environmental harshness on intergroup interaction provides an important avenue for further research.

### Implications

The simulations provide useful insights which may further our understanding of the archaeological record documenting key human transformations taking place 300,000 to 30,000 years ago.

Archaic humans in this period were uniquely pre-adapted to benefit from increasing social tolerance through their capacity to transfer resources to buffer shortfalls, as well as uniquely susceptible to ecological pressures due to their increasing reliance on many different resources (plant and animals resources for food, plant resources for medicines, raw materials (such as flint) for tool production). Simulation modelling explains why specific ecological conditions occurring in certain contexts in Africa after 300,000 years ago, a time of increasing aridification and increasingly variable environments, may have provided the conditions in which elevated selection pressures on intergroup social tolerance might have emerged, leading to the passing of a threshold point beyond which intergroup collaboration became a normal stable state.

Particularly elevated selection pressures would have characterised certain African populations due to a unique combination of body form, ecological context and geography. Gracile or more slightly built humans (*i.e.* emerging modern humans in contrast to more heavily built or ‘robust’ archaic species) have lower energy requirements, and when living in equatorial contexts with high productivity, would exist at higher population densities than robust forms. For this reason, early modern human African populations in many regions would have been buffered from low population densities at which intergroup interactions become impossible. Moreover, such populations would have been uniquely situated within a geographical situation in which large regional scale connectivity was possible. Increased friendly interactions and collaboration between groups would also have enhanced the spread of innovations, regardless of population size or density, thus further enabling greater adaptability to change.

**Fig. 8:**
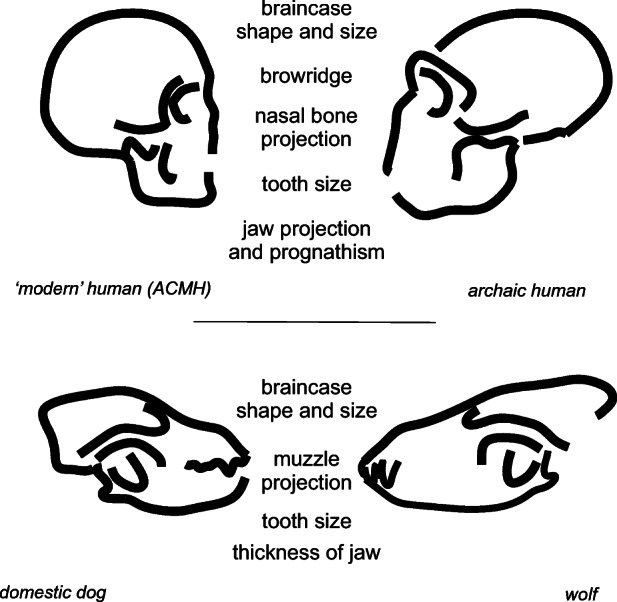
Similarities in cranio-facial changes seen between modern and archaic humans and between dogs and wolves (re-drawn after Theofanopoulou *et al.* 2017)

The model also explains why anatomical features of ‘self-domestication’ associated with increasing tolerance are visible in African population after 300,000 years ago. Self-domestication represents an extreme form of social tolerance, affecting physiology, anatomy and behaviour, most probably through the action of changes in neural crest cells and their effect on the hypothalamic-pituitary-adrenal (HPA) axis (Wilkins *et al.* 2014). Whether the term ‘self-domestication’ is appropriate within human evolution or not (Sánchez-Villagra and van Schaik 2019; Shilton *et al.* 2020), both selective pressures on increased social tolerance and associated anatomical changes provide an explanation for the similarities seen in cranial and facial forms of ACMH compared to archaic species, to changes seen between domestic dogs and wolves (Fig. 8). Whereas domestication occurs through human influence within artificially ‘domesticated’ species, ecological conditions are an influencing factor where increasing levels of intergroup tolerance emerge in ‘wild’ contexts (Pisor and Surbeck 2019) (as described by Hare *et al.* 2012 for bonobos). Although explanations for this process in humans have to date largely drawn on internal social process (Hare 2017; Wrangham 2014, 2019), we argue here that ecological context will have had an important role to play in changing social tolerance and ‘self-domestication’ in humans.

Increased intergroup tolerance thus provides an alternative explanation to that of population size or density (Shennan 2001; Langley *et al.* 2008) for transformations in the occurrence of innovation and cumulative evolution during this period. However, it complements models that link these transformations to increases in population connectivity (*e.g.* Powell *et al.* 2009), and, as demonstrated by our model, increased intergroup tolerance, can also lead to population increase. Moreover, explanations for ‘modern human behaviour’ based on changes brought about through increased intergroup tolerance do not depend on the questionable concept of an increasingly sophisticated cognition (*e.g.* Klein 2000).

### Limitations

Simulation models allow us to test the implications of different scenarios and the relationships between many different variables. Our ABM allows an exploration of how particular processes (human intergroup behaviours) may have been affected by changes in certain variables (ecological changes). Like every model, this simulation cannot represent the full extent of all social interactions among archaic humans. It therefore simplifies some of these aspects to allow us to explore how different strategies of avoidance of other groups or tolerance with the potential for sharing are affected by ecological context.

There is clearly far more to the emergence of hunter-gatherer intergroup tolerance and sharing than emotional dispositions, even though they play an important role (Spikins 2019). For this reason, any model provides us with a starting point and not an end. For example, the level of ecological variability we modelled played only a minor role in influencing the advantages or disadvantages of sharing. However, this may be limited by modelling only ‘simple’ one step interactions, and not accommodating uniquely human emotions such as gratitude (which may play a key role in maintaining generalised reciprocity; Nowak and Roch 2007; Ma *et al.* 2017; Smith *et al.* 2017) nor cultural behaviours such as gift giving (Coward 2015). Future models might address such issues.

### Further Research

The model described here is based at the level of the individual and considered the success of different strategies when compared against each other. This could be developed further in several ways. For example, it would be possible to add an evolutionary component (*i.e.* to enable individuals to evolve across successive generations). This additional complexity would allow questions about evolutionary mechanisms such as group selection to be addressed. Sharing of knowledge could be an additional element which would enable cultural evolution, potentially occurring differently within different groups (Powell *et al.* 2009, Vaesen *et al.* 2016, Lucchesi *et al.* 2020). Differences in memory capacities might also be incorporated into further models (see Cox *et al.*^1999^).

### Research Contribution Within Agent-Based Models in Archaeology

As well as contributing to our understanding of changes taking place 300,000 to 30,000 years ago, particularly the factors influencing the proliferation of regional social networks and increased regional mobility, this research contributes broadly to existing agent-based models which have been used to better understand how resource characteristics influence foraging behaviours. Premo (2005) developed a preliminary model to consider the evolution of food sharing for example. Janssen and Hill (2014), for example, developed a model of foraging behaviour based on actualistic studies of the Hadza, and extended their model to demonstrate that as hunted resources become more clumped the movement distances of hunters reduced (Janssen and Hill 2016). Our model goes beyond resource acquisition and specifically focuses on the relatively under researched topic of food sharing between groups. Models considering intergroup behaviour in this way are rare, with the exception of Santos *et al.* (2015) who explore resource sharing strategies when groups encounter prolific resources in the form of whale carcasses.

## Conclusions

We demonstrate here that external ecological factors may have been more significant in the process of increasing human social tolerance and population connectivity and, in turn, the emergence of ‘modern human behaviour’ than has previously been suggested.

As we have shown, archaic communities would have been particularly sensitive to the effects which ecological changes have on the relative advantages and disadvantages of intergroup social behaviour. They displayed some capacity to share resources between groups and depended on a variety of different resources. The capacity to be tolerant and interact with unfamiliar individuals would have been under particular selection pressures from ~ 300,000 years ago because of the relationship between archaic human resource requirements and ecological changes. Firstly, plant foods and animal resources needed not only for food but also to make tools or medicines are affected by ecological changes, and moreover groups may have depended on access to other essential resources, such as lithic raw materials found outside of their own home ranges. Secondly, ecological factors which influence availability of resources and resource access—including both overall harshness of environment and increasing variability and unpredictably—would have influenced selection pressures on intergroup attitudes and behaviours.

Our model demonstrates that severe resource pressures as well as ecological variability, occurring in environments where population densities are sufficient to allow intergroup interaction, place particular selective pressures on intergroup social tolerance. As a result, particular conditions in Africa after 300,000 years ago may have pushed humans past a turning point in adaptive changes. Once physiological changes passed beyond a certain threshold point, intergroup collaboration may have become the stable state, leading to increasing dependence on varied resources and high levels of social connection, in turn laying the basis for social and cultural transformations.

The effect of ecological changes on intergroup dispositions provides us with an important alternative explanation for changes in social behaviour in recent human evolution. Whilst there has been some understanding of the significance of intergroup collaboration in resilience to ecological changes, this model adds an understanding of how and why intergroup collaboration may have emerged. This approach moves beyond concepts of the progressive development of ‘modern’ cognition towards a more complex relationship between mind, body and social relationships, and moreover provides a means of linking theoretical approaches to ecology and anatomical changes with approaches to social-cognitive changes. Furthermore, by focusing on how ecological context can influence physiological and behavioural changes, we hope to move towards an understanding of social transformations as part of different evolutionary branches and possibilities, rather than a ladder of progression.

## Supplementary Information

ESM 1 (DOCX 372 kb)

## References

[CR1] Agam A, Barkai R (2016). Not the brain alone: the nutritional potential of elephant heads in Paleolithic sites. Quaternary International.

[CR2] Ames CJH, Riel-Salvatore J, Collins BR (2013). Why we need an alternative approach to the study of modern human behaviour. Canadian Journal of Archaeology / Journal Canadien d’Archéologie.

[CR3] Bird DW, Bird RB, Codding BF, Zeanah DW (2019). Variability in the organization and size of hunter-gatherer groups: foragers do not live in small-scale societies. Journal of Human Evolution.

[CR4] Brantingham PJ (2003). A neutral model of stone raw material procurement. American Antiquity.

[CR5] Brooks AS, Yellen JE, Potts R, Behrensmeyer AK, Deino AL, Leslie DE, Ambrose SH, Ferguson JR, d’Errico F, Zipkin AM, Whittaker S, Post J, Veatch EG, Foecke K, Clark JB (2018). Long-distance stone transport and pigment use in the earliest middle stone age. Science.

[CR6] Castellano S, Parra G, Sánchez-Quinto FA, Racimo F, Kuhlwilm M, Kircher M (2014). Patterns of coding variation in the complete exomes of three Neandertals. Proceedings of the National Academy of Sciences USA.

[CR7] Cegielski WH, Rogers JD (2016). Rethinking the role of agent-based modeling in archaeology. Journal of Anthropological Archaeology.

[CR8] Cieri RL, Churchill SE, Franciscus RG, Tan J, Hare B (2014). Craniofacial feminization, social tolerance, and the origins of behavioral modernity. Current Anthropology.

[CR9] Coward F (2015). Scaling up: material culture as scaffold for the social brain. Quaternary International.

[CR10] Cox SJ, Slockin TJ, Steele J (1999). Group size, memory, and interaction rate in the evolution of cooperation. Current Anthropology.

[CR11] Decety J, Norman GJ, Berntson GG, Cacioppo JT (2012). A neurobehavioral evolutionary perspective on the mechanisms underlying empathy. Progress in Neurobiology.

[CR12] d’Errico F, Banks WE (2013). Identifying mechanisms behind Middle Paleolithic and Middle Stone Age cultural trajectories. Current Anthropology.

[CR13] Djindjian F (2012). Is the MP-EUP transition also an economic and social revolution?. Quaternary International.

[CR14] Domínguez-Rodrigo M, Bunn HT, Mabulla AZP, Baquedano E, Uribelarrea D, Pérez-González A (2014). On meat eating and human evolution: a taphonomic analysis of BK4b (Upper Bed II, Olduvai Gorge, Tanzania), and its bearing on hominin megafaunal consumption. Quaternary International.

[CR15] Dyble M, Thompson J, Smith D, Salali GD, Chaudhary N, Page AE, Vinicuis L, Mace R, Migliano AB (2016). Networks of food sharing reveal the functional significance of multilevel sociality in two hunter-gatherer groups. Current Biology.

[CR16] Ekshtain R, Malinsky-Buller A, Ilani S, Segal I, Hovers E (2014). Raw material exploitation around the Middle Paleolithic site of ‘Ein Qashish. Quaternary International.

[CR17] Ekshtain R, Ilani S, Segal I, Hovers E (2017). Local and nonlocal procurement of raw material in Amud Cave, Israel: the complex mobility of Late Middle Paleolithic groups. Geoarchaeology.

[CR18] Faurby S, Silvestro D, Werdelin L, Antonelli A (2020). Brain expansion in early hominins predicts carnivore extinctions in East Africa. Ecology Letters.

[CR19] Féblot-Augustins J, Roebroeks W, Gamble C (1999). Raw material transport patterns and settlement systems in the European Lower and Middle Palaeolithic: continuity, change and variability. The Middle Palaeolithic occupation of Europe.

[CR20] Foley RA (2018). Evolutionary geography and the afrotropical model of hominin evolution. Bulletins et Mémoires de la Société d’Anthropologie de Paris.

[CR21] French JC (2016). Demography and the Palaeolithic archaeological record. Journal of Archaeological Method and Theory.

[CR22] French JC, Fuentes A, Deane-Drummond C (2018). The Palaeolithic archaeological record and the materiality of imagination: a response to J. Wentzel van Huyssteen. Evolution of wisdom: major and minor keys.

[CR23] Galway-Witham J, Cole J, Stringer C (2019). Aspects of human physical and behavioural evolution during the last 1 million years. Journal of Quaternary Science.

[CR24] Gamble C (2009). Human display and dispersal: a case study from biotidal Britain in the Middle and Upper Pleistocene. Evolutionary Anthropology.

[CR25] Greenbaum G, Friesem DE, Hovers E, Feldman MW, Kolodny O (2019). Was inter-population connectivity of Neanderthals and modern humans the driver of the Upper Paleolithic transition rather than its product?. Quaternary Science Reviews.

[CR26] Grove M, Pearce E, Dunbar RIM (2012). Fission-fusion and the evolution of hominin social systems. Journal of Human Evolution.

[CR27] Gurven M, Kaplan H (2007). Longevity among hunter-gatherers: a cross-cultural examination. Population and Development Review.

[CR28] Hardy K (2018). Plant use in the Lower and Middle Palaeolithic: food, medicine and raw materials. Quaternary Science Reviews.

[CR29] Hare B (2017). Survival of the friendliest: *Homo sapiens* evolved via selection for prosociality. Annual Review of Psychology.

[CR30] Hare B, Wobber V, Wrangham R (2012). The self-domestication hypothesis: evolution of bonobo psychology is due to selection against aggression. Animal Behaviour.

[CR31] Hare B, Yamamoto S (2017). Bonobos: unique in mind, brain and behavior.

[CR32] Henshilwood CS, Marean CW (2003). The origin of modern human behavior. Current Anthropology.

[CR33] Hill K, Hurtado AM, Walker RS (2007). High adult mortality among Hiwi hunter-gatherers: implications for human evolution. Journal of Human Evolution.

[CR34] Hill KR, Wood BM, Baggio J, Hurtado AM, Boyd RT (2014). Hunter-gatherer inter-band interaction rates: implications for cumulative culture. PLoS One.

[CR35] Hoffmann DL, Standish CD, García-Diez M, Pettitt PB, Milton JA, Zilhão J, Alcolea-González JJ, Cantalejo-Duarte P, Collado H, de Balbín R, Lorblanchet M, Ramos-Muñoz J, Weniger GC, Pike AWG (2018). U-Th dating of carbonate crusts reveals Neandertal origin of Iberian cave art. Science.

[CR36] Hovers, E. (2018). Continuity and change in research about the Neanderthals in the Levant. *Neanderthal: The Conference, Gibraltar Museum, 13th*, (September 2018).

[CR37] Hublin J-J, Ben-Ncer A, Bailey SE, Freidline SE, Neubauer S, Skinner MM, Bergmann I, Le Cabec A, Benazzi S, Harvati K, Gunz P (2017). New fossils from Jebel Irhoud, Morocco and the pan-African origin of Homo sapiens. Nature.

[CR38] Janssen MA, Hill K (2014). Benefits of grouping and cooperative hunting among Ache hunter–gatherers: insights from an agent-based foraging model. Human Ecology.

[CR39] Janssen, M. A., & Hill, K. (2016). An agent-based model of resource distribution on hunter-gatherer foraging strategies: clumped habitats favor lower mobility but result in higher foraging returns. in J. A., Barceló, & F. Del Castillo, (Eds.). (2016). *Simulating Prehistoric and Ancient Worlds* (pp. 159-174): Dordrecht: Springer.

[CR40] Joordens JCA, d’Errico F, Wesselingh FP, Munro S, de Vos J, Wallinga J, Ankjærgaard C, Reimann T, Wijbrans JR, Kuiper KF, Mücher HJ, Coqueugniot H, Prié V, Joosten I, van Os B, Schulp AS, Panuel M, van der Haas V, Lustenhouwer W, Reijmer JJG, Roebroeks W (2015). *Homo erectus* at Trinil on Java used shells for tool production and engraving. Nature.

[CR41] Kelly, R. L. (2013). *The lifeways of hunter-gatherers: the foraging spectrum*. Cambridge: Cambridge University Press.

[CR42] Kissel M, Fuentes A (2018). “Behavioral modernity” as a process, not an event, in the human niche. Time and Mind.

[CR43] Klein RG (2000). Archeology and the evolution of human behavior. Evolutionary Anthropology.

[CR44] Klein RG (2019). Population structure and the evolution of *Homo sapiens* in Africa. Evolutionary Anthropology.

[CR45] Křivan V, Eisner J (2003). Optimal foraging and predator–prey dynamics III. Theoretical Population Biology.

[CR46] Kutzbach JE, Guan J, He F, Cohen AS, Orland IJ, Chen G (2020). African climate response to orbital and glacial forcing in 140,000-y simulation with implications for early modern human environments. Proceedings of the National Academy of Sciences of the United States of America.

[CR47] Lalueza-Fox C, Rosas A, Estalrrich A, Gigli E, Campos PF, García-Tabernero A, García-Vargas S, Sánchez-Quinto F, Ramírez O, Civit S, Bastir M, Huguet R, Santamaría D, Gilbert MTP, Willerslev E, de la Rasilla M (2011). Genetic evidence for patrilocal mating behavior among Neandertal groups. Proceedings of the National Academy of Sciences USA.

[CR48] Lamb HF, Bates CR, Bryant CL, Davies SJ, Huws DG, Marshall MH, Roberts HM, Toland H (2018). 150,000-year palaeoclimate record from northern Ethiopia supports early, multiple dispersals of modern humans from Africa. Scientific Reports.

[CR49] Langley MC, Clarkson C, Ulm S (2008). Behavioural complexity in Eurasian Neanderthal populations: a chronological examination of the archaeological evidence. Cambridge Archaeological Journal.

[CR50] Lavi N, Friesem DE (2019). Towards a broader view of hunter-gatherer sharing.

[CR51] Layton R, O’Hara S, Bilsborough A (2012). Antiquity and social functions of multilevel social organization among human hunter-gatherers. International Journal of Primatology.

[CR52] Lucchesi S, Cheng L, Janmaat K, Mundry R, Pisor A, Surbeck M (2020). Beyond the group: how food, mates, and group size influence intergroup encounters in wild bonobos. Behavioral ecology: official journal of the International Society for Behavioral Ecology.

[CR53] Ma LK, Tunney RJ, Ferguson E (2017). Does gratitude enhance prosociality? A meta-analytic review. Psychological Bulletin.

[CR54] Marsh AA (2019). The caring continuum: evolved hormonal and proximal mechanisms explain prosocial and antisocial extremes. Annual Review of Psychology.

[CR55] Marwick B (2003). Pleistocene exchange networks as evidence for the evolution of language. Cambridge Archaeological Journal.

[CR56] Mellars P, Mellars P, Boyle K, Bar Yosef O, Stringer C (2007). Rethinking the human revolution: Eurasian and African perspectives. Rethinking the human revolution.

[CR57] Moncel M-H, Schreve D (2016). The Acheulean in Europe: origins, evolution and dispersal. Quaternary International.

[CR58] Nash DJ, Coulson S, Staurset S, Ullyott JS, Babutsi M, Hopkinson L, Smith MP (2013). Provenancing of silcrete raw materials indicates long-distance transport to Tsodilo Hills, Botswana, during the Middle Stone Age. Journal of Human Evolution.

[CR59] Nash DJ, Coulson S, Staurset S, Ullyott JS, Babutsi M, Smith MP (2016). Going the distance: mapping mobility in the Kalahari Desert during the Middle Stone Age through multi-site geochemical provenancing of silcrete artefacts. Journal of Human Evolution.

[CR60] Nowak MA, Roch S (2007). Upstream reciprocity and the evolution of gratitude. Proceedings of the Royal Society B.

[CR61] Owen RB, Muiruri VM, Lowenstein TK, Renaut RW, Rabideaux N, Luo S, Deino AL, Sier MJ, Dupont-Nivet G, McNulty EP, Leet K, Cohen A, Campisano C, Deocampo D, Shen C-C, Billingsley, Mbuthia A (2018). Progressive aridification in East Africa over the last half million years and implications for human evolution. Proceedings of the National Academy of Sciences of the United States of America.

[CR62] Petraglia MD, Breeze PS, Groucutt HS, Rasul NMA, Stewart ICF (2019). Blue Arabia, Green Arabia: examining human colonisation and dispersal models. Geological setting, palaeoenvironment and archaeology of the Red Sea.

[CR63] Pisor AC, Surbeck M (2019). The evolution of intergroup tolerance in nonhuman primates and humans. Evolutionary Anthropology.

[CR64] Potts R (2013). Hominin evolution in settings of strong environmental variability. Quaternary Science Reviews.

[CR65] Potts R, Behrensmeyer AK, Faith JT, Tryon CA, Brooks AS, Yellen JE, Deino AL, Kinyanjui R, Clark JB, Haradon CM, Levin NE, Meijer HJM, Veatch EG, Owen RB, Renaut RW (2018). Environmental dynamics during the onset of the Middle Stone Age in eastern Africa. Science.

[CR66] Powell A, Shennan S, Thomas MG (2009). Late Pleistocene demography and the appearance of modern human behavior. Science.

[CR68] Premo, L. S. (2005). Patchiness and prosociality: an agent-based model of Plio/Pleistocene hominid food sharing. In P. Davidsson, B. Logan, & K. Takadama (Eds.), *Multi-agent and multi-agent-based simulation. MABS 2004* (pp. 210–224). Lecture Notes in Computer Science (vol. 3415). Berlin, Heidelberg: Springer.

[CR67] Premo LS (2006). Exploratory agent-based models: towards an experimental ethnoarchaeology, *Digital discovery: exploring new frontiers in human heritage*. Computer Applications in Archaeology.

[CR69] Prüfer K, Racimo F, Patterson N, Jay F, Sankararaman S, Sawyer S, Heinze A, Renaud G, Sudmant PH, de Filippo C, Li H, Mallick S, Dannemann M, Fu Q, Kircher M, Kuhlwilm M, Lachmann M, Meyer M, Ongyerth M, Siebauer M, Theunert C, Tandon A, Moorjani P, Pickrell J, Mullikin JC, Vohr SH, Green RE, Hellmann I, Johnson PLF, Blanche H, Cann H, Kitzman JO, Shendure J, Eichler EE, Lein ES, Bakken TE, Golovanova LV, Doronichev VB, Shunkov MV, Derevianko AP, Viola B, Slatkin M, Reich D, Kelso J, Pääbo S (2014). The complete genome sequence of a Neanderthal from the Altai Mountains. Nature.

[CR70] Railsback SF, Grimm V (2019). Agent-based and individual-based modeling: a practical introduction.

[CR71] Richter D, Grün R, Joannes-Boyau R, Steele TE, Amani F, Rué M, Fernandes P, Raynal JP, Geraads D, Ben-Ncer A, Hublin JJ, McPherron SP (2017). The age of the hominin fossils from Jebel Irhoud, Morocco, and the origins of the Middle Stone Age. Nature.

[CR72] Ríos L, Rosas A, Estalrrich A, García-Tabernero A, Bastir M, Huguet R, Pastor F, Sanchís-Gimeno JA, de la Rasilla M (2015). Possible further evidence of low genetic diversity in the El Sidrón (Asturias, Spain) Neandertal group: congenital clefts of the atlas. PLoS One.

[CR73] Ríos L, Kivell TL, Lalueza-Fox C, Estalrrich A, García-Tabernero A, Huguet R, Quintino Y, de la Rasilla M, Rosas A (2019). Skeletal anomalies in the Neandertal family of El Sidrón (Spain) support a role of inbreeding in Neandertal extinction. Scientific Reports.

[CR74] Rito T, Vieira D, Silva M, Conde-Sousa E, Pereira L, Mellars P, Richards MB, Soares P (2019). A dispersal of *Homo sapiens* from southern to eastern Africa immediately preceded the out-of-Africa migration. Scientific Reports.

[CR75] Robinson EJH, Barker JL (2017). Inter-group cooperation in humans and other animals. Biology Letters.

[CR76] Romanowska I, Crabtree SA, Harris K, Davies B (2019). Agent-based modeling for archaeologists: Part 1 of 3. Advances in Archaeological Practice.

[CR77] Sánchez-Villagra MR, van Schaik CP (2019). Evaluating the self-domestication hypothesis of human evolution. Evolutionary Anthropology.

[CR78] Santos JI, Pereda M, Zurro D, Álvarez M, Caro J, Galán JM, Briz i Godino I (2015). Effect of resource spatial correlation and hunter-fisher-gatherer mobility on social cooperation in Tierra del Fuego. PLoS One.

[CR79] Sapolsky, R. M. (2017). *Behave: the biology of humans at our best and worst*Penguin.

[CR80] Scerri EML, Thomas MG, Manica A, Gunz P, Stock JT, Stringer C (2018). Did our species evolve in subdivided populations across Africa, and why does it matter?. Trends in Ecology & Evolution.

[CR81] Shennan S (2001). Demography and cultural innovation: a model and its implications for the emergence of modern human culture. Cambridge Archaeological Journal.

[CR82] Shilton D, Breski M, Dor D, Jablonka E (2020). Human social evolution: self-domestication or self-control?. Frontiers in Psychology.

[CR83] Sikora M, Seguin-Orlando A, Sousa VC, Albrechtsen A, Korneliussen T, Ko A, Rasmussen S, Dupanloup I, Nigst PR, Bosch MD, Renaud G, Allentoft ME, Margaryan A, Vasilyev SV, Veselovskaya EV, Borutskaya SB, Deviese T, Comeskey D, Higham T, Manica A, Foley R, Meltzer DJ, Nielsen R, Excoffier L, Mirazon Lahr M, Orlando L, Willerslev E (2017). Ancient genomes show social and reproductive behavior of early Upper Paleolithic foragers. Science.

[CR84] Simon MH, Ziegler M, Bosmans J, Barker S, Reason CJC, Hall IR (2015). Eastern South African hydroclimate over the past 270,000 years. Scientific Reports.

[CR85] Smith A, Pedersen EJ, Forster DE, McCullough ME, Lieberman D (2017). Cooperation: the roles of interpersonal value and gratitude. Evolution and Human Behavior.

[CR86] Spikins P, Levi N, Friesem D (2019). Sharing and inclusion: a socio-emotional model of generosity, trust and response to vulnerability in the distant past. Towards a broader view of hunter-gatherer sharing.

[CR87] Spikins, P. (2021). *Hidden depths: the palaeolithic origins of our most human emotions*. White Rose University Press.

[CR88] Spikins P, Needham A, Wright B, Dytham C, Gatta M, Hitchens G (2019). Living to fight another day: the ecological and evolutionary significance of Neanderthal healthcare. Quaternary Science Reviews.

[CR89] Spinapolice EE (2012). Raw material economy in Salento (Apulia, Italy): new perspectives on Neanderthal mobility patterns. Journal of Archaeological Science.

[CR90] Stringer C, Galway-Witham J (2017). Palaeoanthropology. On the origin of our species Nature.

[CR91] Tan J, Hare B (2013). Bonobos share with strangers. PLoS One.

[CR92] Tan J, Ariely D, Hare B (2017). Bonobos respond prosocially toward members of other groups. Scientific Reports.

[CR93] Theofanopoulou, C., Andirko, A., & Boeckx, C. (2018). Oxytocin and vasopressin receptor variants as a window onto the evolution of human prosociality. In *bioRxiv* (p. 460584). https://doi.org/10.1101/460584.

[CR94] Theofanopoulou C, Gastaldon S, O’Rourke T, Samuels BD, Martins PT, Delogu F, Alamri S, Boeckx C (2017). Self-domestication in *Homo sapiens*: insights from comparative genomics. PLoS One.

[CR95] Thomas J, Kirby S (2018). Self domestication and the evolution of language. Biology and Philosophy.

[CR96] Thornton A, McAuliffe K (2006). Teaching in wild meerkats. Science.

[CR97] Timmermann A, Friedrich T (2016). Late Pleistocene climate drivers of early human migration. Nature.

[CR98] Trinkaus E (2018). An abundance of developmental anomalies and abnormalities in Pleistocene people. Proceedings of the National Academy of Sciences USA.

[CR99] Vaesen K, Collard M, Cosgrove R, Roebroeks W (2016). Population size does not explain past changes in cultural complexity. Proceedings of the National Academy of Sciences USA.

[CR100] White JW, Rassweiler A, Samhouri JF, Stier AC, White C (2014). Ecologists should not use statistical significance tests to interpret simulation model results. Oikos.

[CR101] Wiessner P, Salter FK (2002). Taking the risk out of risky transactions: a forager’s dilemma. Risky transactions: trust, kinship, and ethnicity.

[CR102] Wilkins AS, Wrangham RW, Tecumseh Fitch W (2014). The “Domestication Syndrome” in mammals: a unified explanation based on neural crest cell behavior and genetics. Genetics.

[CR103] Wittig RM, Crockford C, Deschner T, Langergraber KE, Ziegler TE, Zuberbühler K (2014). Food sharing is linked to urinary oxytocin levels and bonding in related and unrelated wild chimpanzees. Proceedings of the Royal Society B.

[CR104] Wrangham, R. (2014). *Did Homo sapiens self-domesticate?* https://carta.anthropogeny.org/events/sessions/did-homo-sapiens-self-domesticate.

[CR105] Wrangham RW (2019). Hypotheses for the evolution of reduced reactive aggression in the context of human self-domestication. Frontiers in Psychology.

[CR106] Zilhão J, Angelucci DE, Badal-García E, d’Errico F, Daniel F, Dayet L (2010). Symbolic use of marine shells and mineral pigments by Iberian Neandertals. Proceedings of the National Academy of Sciences USA.

