# Supplementary material for: Theoretical and Methodological Approaches to Ecological Changes, Social Behaviour and Human Intergroup Tolerance 300,000 to 30,000 BP
Source: J Archaeol Method Theory. 2021 Feb 3;28(1):53–75. doi: 10.1007/s10816-020-09503-5 (PMC7891228; doi:10.1007/s10816-020-09503-5)
Supplement: Supplementary file 1 — (DOCX 372 kb) [file 10816_2020_9503_MOESM1_ESM.docx]

**Supplementary Information for Spikins *et al*. “Theoretical and methodological approaches to ecological changes, social behaviour and human intergroup tolerance 300,000 to 30,000 BP”**

**Model details**

**Hunting**: (see supplementary Figure S1 for a graphical outline of the protocol). Move steps during hunting are executed in series. First, an individual is selected at random from the whole population. If that individual is of age 10 or above, they move a random distance drawn from a uniform distribution [0,1] in a random direction. If the move would take them more than 7 grid units from their home, they immediately return home (from where they can start a new hunting foray next time they are selected). At the end of the move they hunt. Hunting success is set by the animal density of the current landscape square (more success in higher animal density populations (pop) with a simple linear probability of pop/100). With a successful hunt the local animal population level is reduced by 1 and the group's food stock increases. This process is repeated 30 x current population size times, selecting a random individual each time. This means that each individual does not take a fixed number of move steps but will make a number of steps that is Poisson distributed with a mean of 30.


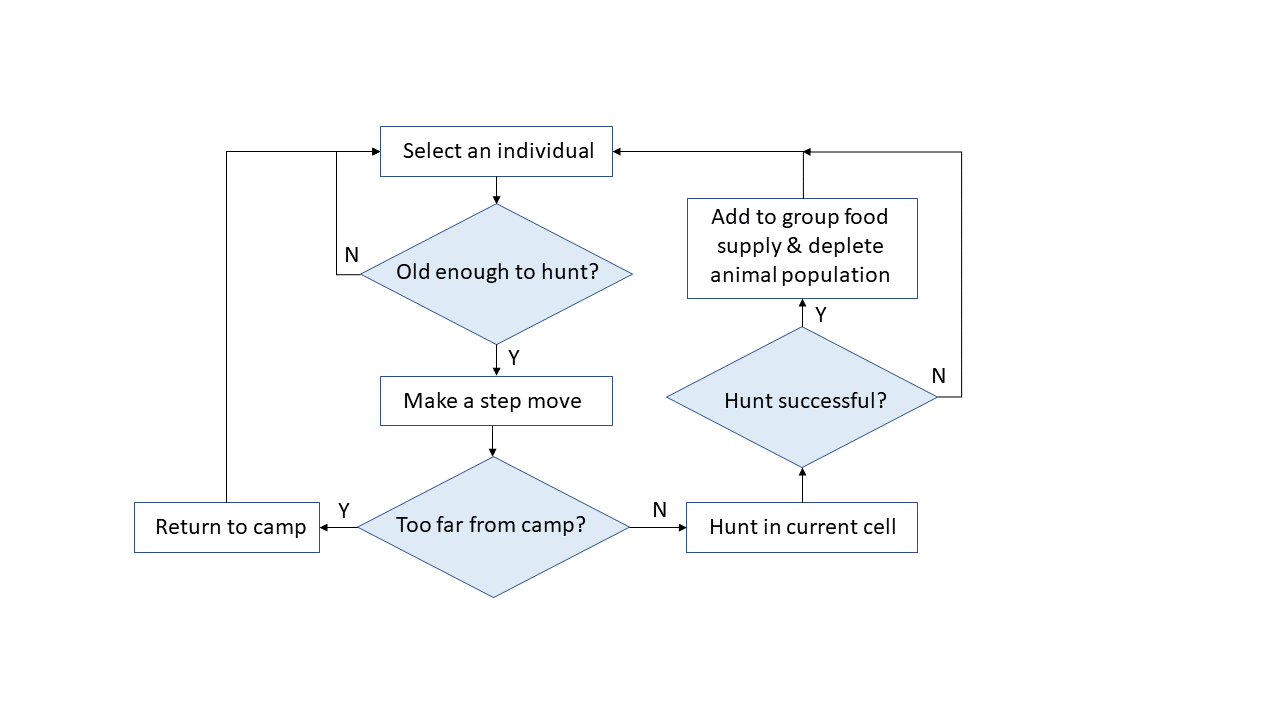


**Figure S1** Workflow for hunting protocol

**Intergroup interactions:** Pairs of groups are selected at random and the Euclidean distance between group foci calculated. There is a declining probability of interaction with distance such that groups 25 grid units apart will meet 75% of the time while those more than 50 grid units apart never interact. This leads to about 5% of groups having interactions in a season. When ‘tolerant’ groups meet they may transfer resources. If one group has a deficit of resources (i.e. not enough for subsistence for all members of the group) and the other has a surplus (i.e. more than enough for both subsistence and one birth) then 10 units of resource are passed from one group to the other.

**Starvation and relocation:** When the pool of resources that a group gains during hunting is insufficient for maintenance starvation may occur. Each individual in a group has a probability of: (group size - food available) / group size of death due to starvation. For example, if there was enough food for half of the group then all members would have a 0.5 mortality probability applied in this phase. If any starvation occurs, the group will look for a new location and move their base camp (group focus). A random location within 14 grid units of the current group focus is selected. The group will shift focus and all individuals will move there if the local food level (animal population in the alternative location cell) is higher than that in the current location.

**Group fission and relocation:** Groups reaching a size of 50 split into two. Each individual in the parent group is assigned at random, with even probability, to one of the two daughter groups. At the fission event one of the two daughter groups, determined at random, retains the parent group’s focus (‘base camp’ location). The other group focus, and all individuals in this daughter group, moves to a random location within 14 grid unit points of the previous focus. To ensure a good starting location is selected, the new focus must be located in a landscape cell with a higher animal population level than that in the original location, representing some scouting of the location before moving.

**Animal population growth:** Each landscape grid cell supports an independent population of animals which is similar to Janssen and Hill (2014; 2016). Animal populations follow logistic growth with an *r* of 0.1 and *K* of 100 using this equation: pop_t+1_ = pop_t_ + (pop_t_ * *r* * (1 - pop_t_/*K*)).

If any animal population falls below 1 it is reset to 1 to prevent local extinction. Unlike Janssen and Hill (2014) there is no movement of animals between grid cells. Animal populations are not restricted to integers.

**Sensitivity analysis**

**Group size:** We collected data on the size of groups at the end of simulations (time 1000). The mean group size is ~23 individuals (which fits with records of modern foraging populations, see Kelly 2013), the distribution of group sizes is shown in Figure S2. Few groups are small and close to dissolution (size 4) and few groups are large and close to fission (size 50).

*
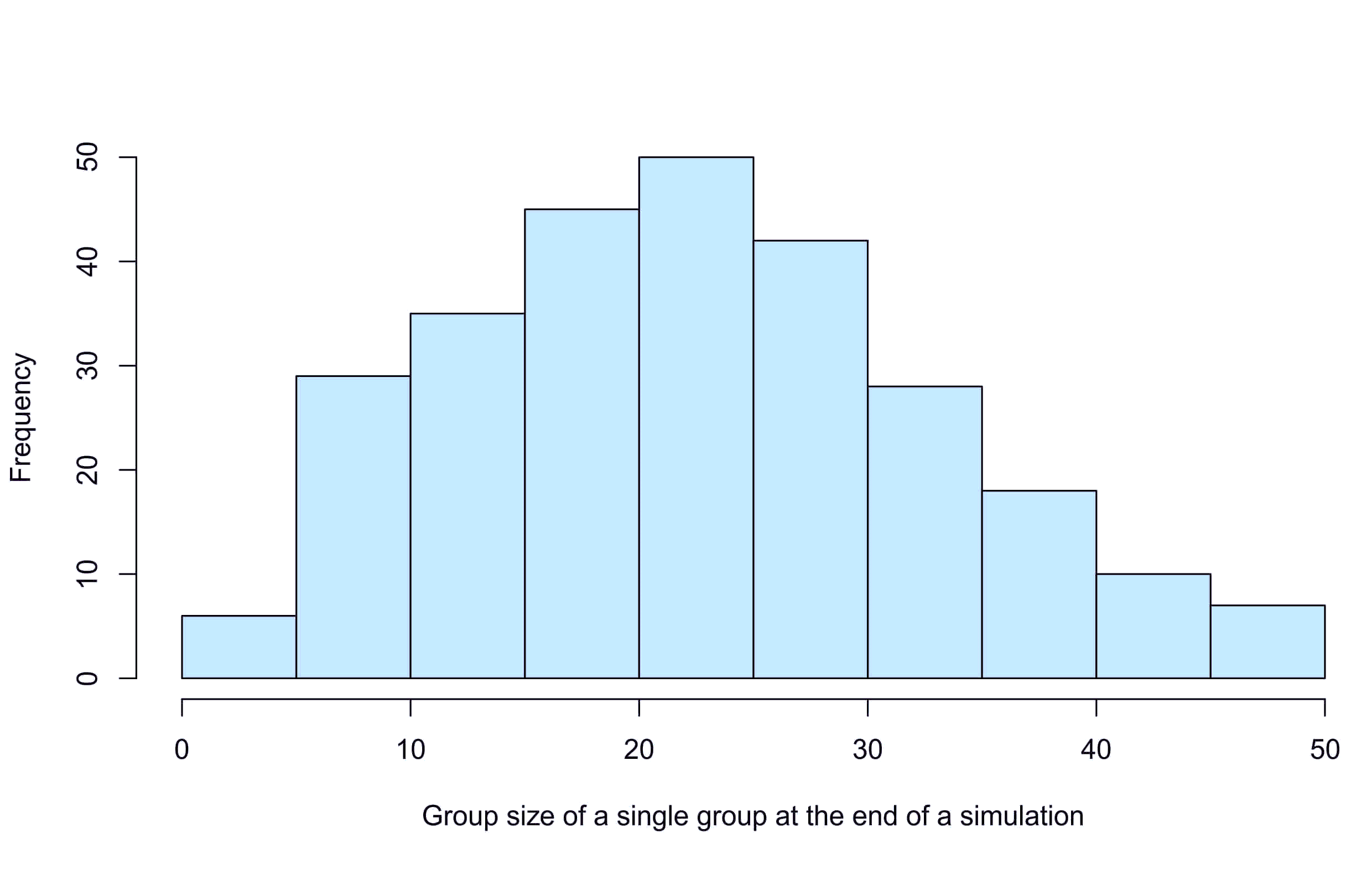
***Figure S2** The distribution of group sizes at the end of simulations

**References cited**

Janssen, M. A., & Hill, K. (2014). Benefits of Grouping and Cooperative Hunting Among Ache Hunter Gatherers: Insights from an Agent-Based Foraging Model. *Human Ecology*, *42*(6), 823–835.

Kelly, R.L. (2013). *The Lifeways of Hunter-Gatherers: The Foraging Spectrum.* Cambridge: Cambridge University Press.
